# Simple modeling of FtsZ polymers on flat and curved surfaces: correlation with experimental in vitro observations

**DOI:** 10.1186/1757-5036-2-8

**Published:** 2009-10-22

**Authors:** Alfonso Paez, Pablo Mateos-Gil, Ines Hörger, Jesús Mingorance, Germán Rivas, Miguel Vicente, Marisela Vélez, Pedro Tarazona

**Affiliations:** 1 Departamento de Física Teórica de la Materia Condensada, C-V-6a Universidad Autónoma de Madrid, Madrid E-28049, Spain; 2 Instituto Nicolás Cabrera de Ciencia de Materiales, C-XVI-4a, Universidad Autónoma de Madrid, Madrid E-28049, Spain; 3 Unidad de Investigación y Servicio de Microbiología, Hospital Universitario La Paz, Paseo de La Castellana, 261, Madrid, E-28046, Spain; 4 Centro de Investigaciones Biológicas, CSIC, Ramiro de Maeztu 9, E-28040 Madrid, Spain; 5 Centro Nacional de Biotecnología, CSIC, Campus de Cantoblanco, C/Darwin n 3, Madrid E-28049, Spain; 6 Instituto de Catálisis y Petroleoquímica, CSIC C/Marie Curie, 2, Cantoblanco, Madrid, E-28049, Spain; 7 Instituto Madrileño de Estudios Avanzados en Nanociencia (IMDEA-Nanociencia) Facultad de Ciencias, C-IX-3a Cantoblanco, Madrid, E-28049, Spain

## Abstract

FtsZ is a GTPase that assembles at midcell into a dynamic ring that constricts the membrane to induce cell division in the majority of bacteria, in many archea and several organelles. In vitro, FtsZ polymerizes in a GTP-dependent manner forming a variety of filamentous flexible structures. Based on data derived from the measurement of the in vitro polymerization of Escherichia coli FtsZ cell division protein we have formulated a model in which the fine balance between curvature, flexibility and lateral interactions accounts for structural and dynamic properties of the FtsZ polymers observed with AFM. The experimental results have been used by the model to calibrate the interaction energies and the values obtained indicate that the filaments are very plastic. The extension of the model to explore filament behavior on a cylindrical surface has shown that the FtsZ condensates promoted by lateral interactions can easily form ring structures through minor modulations of either filament curvature or longitudinal bond energies. The condensation of short, monomer exchanging filaments into rings is shown to produce enough force to induce membrane deformations.

PACS codes: 87.15.ak, 87.16.ka, 87.17.Ee

## 1 Background

Cell and organelle division involve a force generation step to produce the final mechanical division into two independent structures. Eukaryotic cells rely on the formation of a contractile ring based on actin and myosin [1] whereas bacteria, archea and chloroplasts depend on the ring formed by FtsZ [2-4]. Actin and myosin contractile and force generation mechanism has been associated to an ATP dependent sliding mechanism [5,6], but the force generation mechanism of the FtsZ ring remains unknown. Contractile molecular motors have not been described in bacteria and recent in vitro experiments indicate that FtsZ alone can form rings that deform tubular liposomes in a GTP-dependent manner [7], indicating that the capacity to form a contractile ring on a cylindrical surface is embedded in the FtsZ monomers undergoing dynamic polymerization in the presence of GTP.

Different models derived from available in vitro polymerization data have attempted to provide an explanation for the mechanism underlying the force generation, but no consensus has yet been reached. Some force generating mechanisms are based on filament condensation induced by lateral interactions [8-11] while others are based on curvature induced effects associated with GTP binding and hydrolysis [12-17]. Unfortunately, comparison of the simulations with data available from in vitro polymerization assays in bulk solution does not provide detailed enough information to unambiguously identify the prevailing mechanism.

AFM images of FtsZ formed under GTP hydrolyzing conditions show individual filaments exchanging monomers and dynamically reorganizing into different shaped condensed aggregates [18]. In this work, that high resolution structural and dynamic information has been used to develop a comprehensive theoretical model. We present a fine triangular lattice model inspired by the experiments that accounts for and simulates the complex polymorphic filament aggregates observed experimentally. Four parameters are included as basic traits of the FtsZ filaments: the polymeric (GTP-mediated) monomer-monomer bonding energy, the flexibility and preferential angle of the bond between monomers, and the lateral attraction between neighboring filaments.

We have used these monomer-monomer interactions to explore aggregate formation on a cylindrical surface. We found that a subtle modulation of filament curvature or longitudinal bond energy, together with the presence of condensing lateral interactions, easily gives rise to ring like dynamic filament aggregates capable of generating a constriction force.

## 2 Results

### 2.1 Experiments: Atomic Force Microscopy images

Atomic force microscope images of *E. coli *FtsZ polymerized in the presence of GTP adsorbed on mica were taken with a microscope from Nanotec Electrónica (Madrid, Spain) operated in the jump mode [19]. Silicon nitride tips (DI Instruments) with a force constant of 0.05 *N/m *were used. A drop of the solution with the E. coli FtsZ polymers (formed upon addition of 10 *mM *GTP to FtsZ protein solutions in Tris 50 *mM*, *pH*7, 0.5 *M KCl*, and 5 *mM MgCl*_2 _buffer) was incubated over freshly cleaved mica. After incubating for a few minutes, samples were washed extensively with working buffer and imaged under buffer solution containing 1 *mM *GTP. E. coli FtsZ was purified by the calcium-induced precipitation method following the procedure described previously [20]

### 2.2 Theory: Model building from experimental data

Kinetic and lattice models of FtsZ polymers proposed until now [8,9] have been built based on data obtained from bulk solution polymerization experiments or electron microscopy images of fixed filaments. Some of the kinetic models [12,13] aimed mainly at understanding the cooperative polymerization behavior observed in dilute solution [21-24]. Others have also proposed force generating mechanisms [8,9,14-16], but model refinement has been limited by the lack of a detailed combined description of the structure and dynamic rearrangement of individual filaments. The model presented here springs from new experimental data describing the self-organization of individual filaments on a two dimensional surface under buffer conditions in which GTP hydrolysis takes place. Filaments exchange monomers and aggregate into interconnecting structures of different shapes depending on the amount of protein on the surface [18]. The description of the shapes, dimensions and densities of the aggregates provides enough information to model filaments from the essential monomer-monomer interactions. This different level of representation is both simple in its parametric space and very detailed because all the polymerization, cyclation, and aggregation processes come out directly from the monomer-monomer interactions established under the constrains of a crowded and confined two-dimensional system.

**Figure 1:**
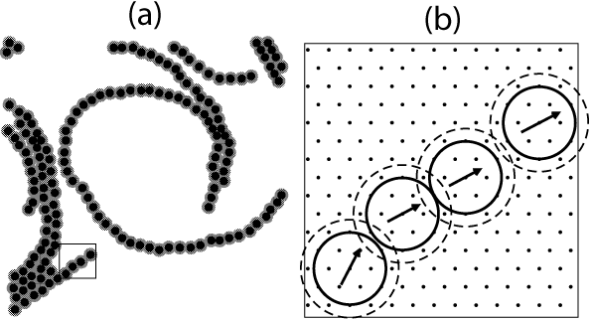
**Schematic representation of the model**. Left panel illustrates a low magnification drawing of monomers on the triangular lattice forming filaments with different degrees of aggregation and curvature. The right panel shows a close look at the region enclosed in a rectangle in the right panel illustrating the 1.5 *nm *triangular lattice spacing, the vector used to define the monomer orientation, from the minus (T7 loop-region) to the plus end (GTP binding site), and the region around the excluding core where attractive interactions are present. The stronger longitudinal *bonding interaction U*_*b *_applies when the orientations of two consecutive monomers is aligned within 30 degrees (see Additional file 1 for more details).

**Figure 2:**
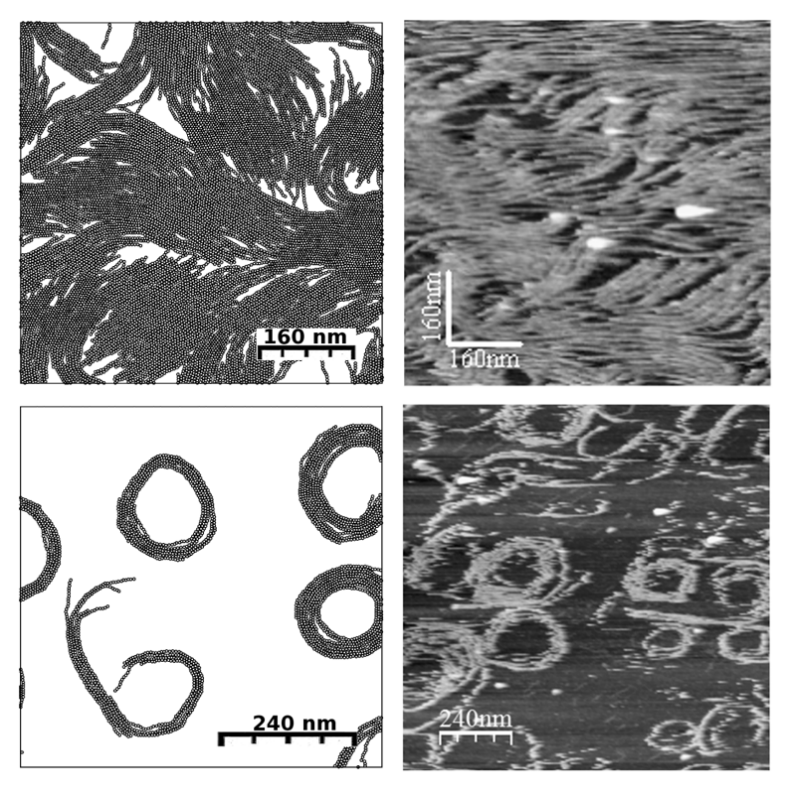
**Experiments versus simulations**. The right hand column shows AFM images of filaments on a mica surface at different protein densities. The left hand column shows the distribution of monomer aggregate obtained from running the simulation at the corresponding equivalent surface protein densities. See text for explanation.

To model the FtsZ filaments adsorbed on mica, the positions of the individual FtsZ protein monomers are restricted to the sites of a two dimensional triangular lattice separated by a distance of 1.5 *nm*. The monomers on the substrate have an axis running from their *minus end *(T7 loop-region) to their *plus end *(GTP binding site) parallel to the planar or cylindrical surface. The axis orientation is limited to a set of 24 directions. This alignment is hardly distinguished at a resolution equivalent to the observed in the AFM images (Fig. 1(a)), but is clearly seen in a detailed representation of a few monomers along a filament as presented in (Fig. 1(b)). This fine-grained lattice was found to be enough to accommodate the spontaneous curvature and flexibility of the filaments, previously proven to be important to explain the behavior of stabilized FtsZ filaments (with no observed monomer exchange) formed in the presence of *GDP - AlF*_3 _[10]. A finer mesh would significantly increase the computational cost of the simulation without adding relevant information for the comparison with AFM experiments. On the other hand, a coarser model, although more accessible computationally, is not able to account for the experimental observations. A previously described coarse square-lattice representations that also considered dynamic monomer exchange ([8,9]) using a cell size comparable to the diameter of the protein monomer, 4.5 *nm*, and allowing only 4 possible monomer orientations was not enough to accommodate the curvature and flexibility of the filaments observed experimentally (see Fig. 2)

Three parameters tune the properties of the longitudinal bond between the monomers forming the filaments. The bond energy -*U*_*b *_can be modulated by the additional energetic cost of deviating contacting monomers from linearity either clockwise (*U*_+_) or anticlockwise (*U*_-_). No penalization for curving on either direction, *U*_+ _= *U*_- _= 0, would represent very flexible filaments with no preferential curvature, whereas large symmetric values would produce very stiff and straight filaments. An additional fourth energy parameter in the model describes the lateral attractive interactions -*U*_*a*_, between the monomers [25].

Monte Carlo (MC) simulations run at different protein densities explore the shapes and rearrangements adopted by the filaments. A full description of the model and the extrapolation to a cylindrical surface is presented in [25] and in Additional file 1.

### 2.3 Comparison with experiments

#### Two dimensional flat surface

The interaction parameters in the fine-grained lattice model can be tuned to reproduce the experimental AFM results (see [25] and Additional file 1). Comparison of the shape and length of the filaments at low protein coverage with the structures obtained from the model allows calibrating the different energy values. The set of values that provide a good description of the observed structures are: a bond energy *U*_*b *_= 12(± 2)*kT *≈ 7.2 *kcal/mol*, a small left-bent penalization *U*_- _= 0.5(± 0.5)*kT*, *U*_+ _= *U*_*b *_to cancel the right-bent and a lateral attraction *U*_*a *_= 0.5(± 0.1)*kT *≈ 0.3 *kcal/mol*. This longitudinal bond energy is comparable to available experimental estimates [26]. The lateral interaction is 20 times weaker than the polymeric bond, explaining the hierarchical structure observed: monomers forming filaments that bundle to form aggregates. A more complete description of the energy calibration procedure is presented in [25] and in Additional file 1. Fig. 2 shows that the same set of parameters used at different protein coverage gives a good match between theory and experiments over the whole range of observed structures, from isolated spiral clusters at low protein coverage to complex percolating (interconnecting) networks at high protein coverage.

**Figure 3:**
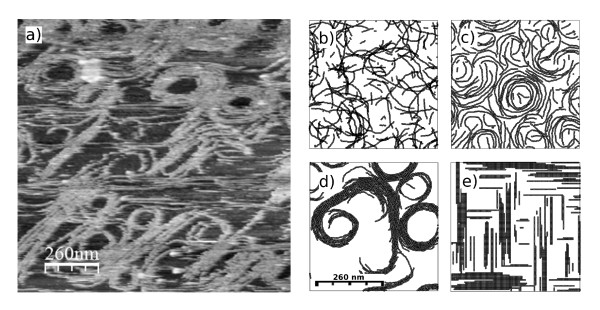
**Experiments versus simulations: incomplete parameters**. The AFM image (a) is compared with equilibrated snapshots of MC simulations of our fine-grained lattice model with incomplete sets of interaction effects. Panel (b) shows filaments formed when the simulation does not include lateral interactions nor excluded volume effects (i.e. isodesmic ideal polymers). Panel (c) comes from a simulation without lateral interactions (*U*_*a *_= 0), to be compared with the snapshot in panel (d) using the complete set of model interactions. Panel (e) corresponds to the coarser square-lattice model presented by Lan et al. [9] at the same protein coverage of a planar surface as in all the other panels.

The quantitative representation of curvature, longitudinal bonding and lateral interactions of the filaments considered in this integrative model correspond to the minimal set of parameters needed to reproduce the rich polymorphism observed in the high resolution AFM images. All these effects had been considered separately in previous FtsZ models [8-10,12-14,16], but they had never been combined to explore their joint effect. Fig. 3 illustrates the aggregates formed when some of the monomer-monomer interactions considered here are turned off, as implicitly done in previous theoretical models. Each panel corresponds to a typical MC snapshot for the same protein coverage as in Fig. 3(a). Ideal polymer treatments, described in terms of the reaction rates for the growth, splitting and cyclation of the filaments, e.g. [14], would describe a version of our model in which repulsive excluded area and lateral interactions are eliminated. The MC result for *this ideal polymer *situation is presented in Fig. 3(b) with overlapping filaments and no tendency to form condensed aggregates. The inclusion of excluded area effects but not lateral interactions between monomers produces the structures in panel (c), still unable to reproduce the experimental observations. The formation of the observed dense aggregates only occurs in the presence of a lateral attraction between FtsZ filaments (panel (d)).

Fig. 3(b-d), with the same longitudinal bond energy and preferential angle between monomers, shows that the excluded area effects and lateral attractions act cooperatively to stabilize longer filaments. Some reaction rate models represent the cooperative polymerization in terms of effective "bundling coefficients" [13]. However those coefficients would depend on protein coverage since the packing constraints shift the energy/entropy balance between the different structures. Our model includes all the "bundling effects" through the value of a unique parameter *U*_*a *_= 0.5 *kT*, the lateral interaction, that accurately accounts for the bundling and observed cooperative lengthening effect independently of protein density, as shown in Fig. 3

#### Cooperative polymerization

**Figure 4:**
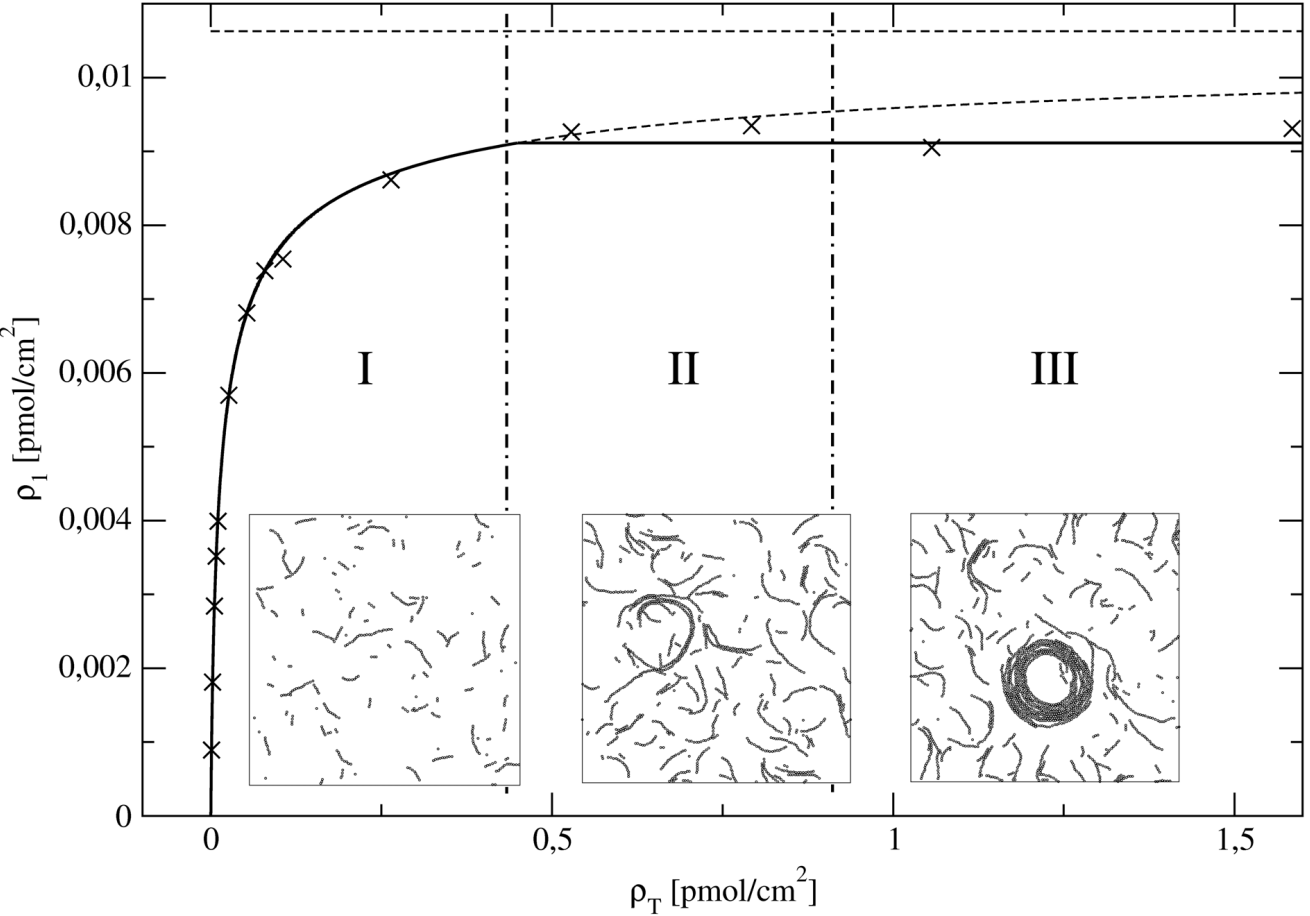
**Cooperative polymerization behavior**. The concentration of unpolymerized protein monomers *ρ*_1 _is plotted as a function of the total monomer concentration *ρ*_T_. The × symbols correspond to averages over equilibrated MC samplings, the short-dashed line (equal to the continuous line in the low density region I, is the isodesmic (ideal polymer) theoretical prediction for our model, which saturates to the long-dashed horizontal line (isodesmic CMC). The full horizontal line over regions II and III represents the true CMC due to the cooperative polymerization induced by the lateral attractions and the core repulsion. The insets show typical snapshots of the system in the isodesmic (I), non-ideal polymers (II) and condensed (III) regions, with protein coverage of the surface from 0.26 to 1.06 *picomol/cm*^2^. The interaction parameters are those corresponding to the wild type FtsZ on mica *U*_*a *_= 0.5 *kT*, *U*_*b *_= *U*_+ _= 12 *kT *and *U*_- _= 0.5 *kT *([25] and Additional file 1)

A polymerization curve presenting the amount of unpolymerized FtsZ, *ρ*_*1*_ as a function of the total protein concentration can be estimated from the model (see Fig.4). The curve agrees with previous experimental results in bulk solution that show a cooperative polymerization behavior. The insets are snapshots of the structures obtained along MC simulations at equilibrium for low surface coverage (between 0.26 and 1.06 *picomol/cm*^2^) of the FtsZ protein.

The effective bundling coefficients is included here through the unique parameter *U*_*a *_= 0.5 *kT*, the lateral interaction, and accurately accounts for the bundling and observed cooperative lengthening effect at any protein density. Below *ρ*_*T *_≈ 0.4 *picomol/cm*^2 ^the model follows an ideal (isodesmic) behavior. The extrapolation (broken line) leads to the upper bound of unpolymerized protein concentration of *ρ*_*o *_≈ 0.0106 *picomol/cm*^2^, at which the polymerized fraction would fill the system. The cooperativity induced by the lateral attractions, balanced by packing constraints and filament curvature, produces the saturation of *ρ*_*o *_at 0.0091 *picomol/cm*^2 ^(86% of its ideal limit). The snapshots in the insets are typical configurations of our model in the regions I (ideal polymers), II (non-ideal polymers), and III (condensed aggregates). Notice that from the rough information given by *ρ*_*o*_(*ρ_T_*) we could not see any difference between the regimes II and III, although they clearly differ in the degree of condensation. In region II the interaction between filaments leads to the formation of longer filaments stabilized by lateral interactions, whereas in region III higher order roll-shaped aggregates with a well defined typical size are present. The AFM experimental information, equivalent to that given in the insets, provides more structural detail than any global measurement of the FtsZ polymerized fraction. It is able to clearly distinguish the condensation of clusters from the mere cooperative polymerization, thus providing experimental support to the predictions of this simple but accurate model of FtsZ polymerization. To illustrate the contribution of this model to previously existing ones, the square-lattice described in a very recent work [9] is used to simulate filaments formed on a planar surface of similar size and protein coverage than the ones observed experimentally (Fig. 3(e)). The square-lattice model includes a very strong longitudinal bond (17 *kT*) and a weaker lateral attraction (0.2 *kT*) between parallel filaments and neglects any flexibility or curvature of the filaments. Furthermore, the lateral attraction is only considered between filaments with the same orientation. These assumptions lead to the formation of isolated elongated drop condensates of different orientation interacting only through excluded area effects very different from the percolating and open polymorphic structures observed experimentally.

The inclusion of curvature and flexibility, together with the lateral interactions, is therefore crucial for the assembly of aggregates with the global structures and filament sharing observed experimentally.

### 2.4 Extrapolation to a cylindrical surface

#### Z-ring formation

**Figure 5:**
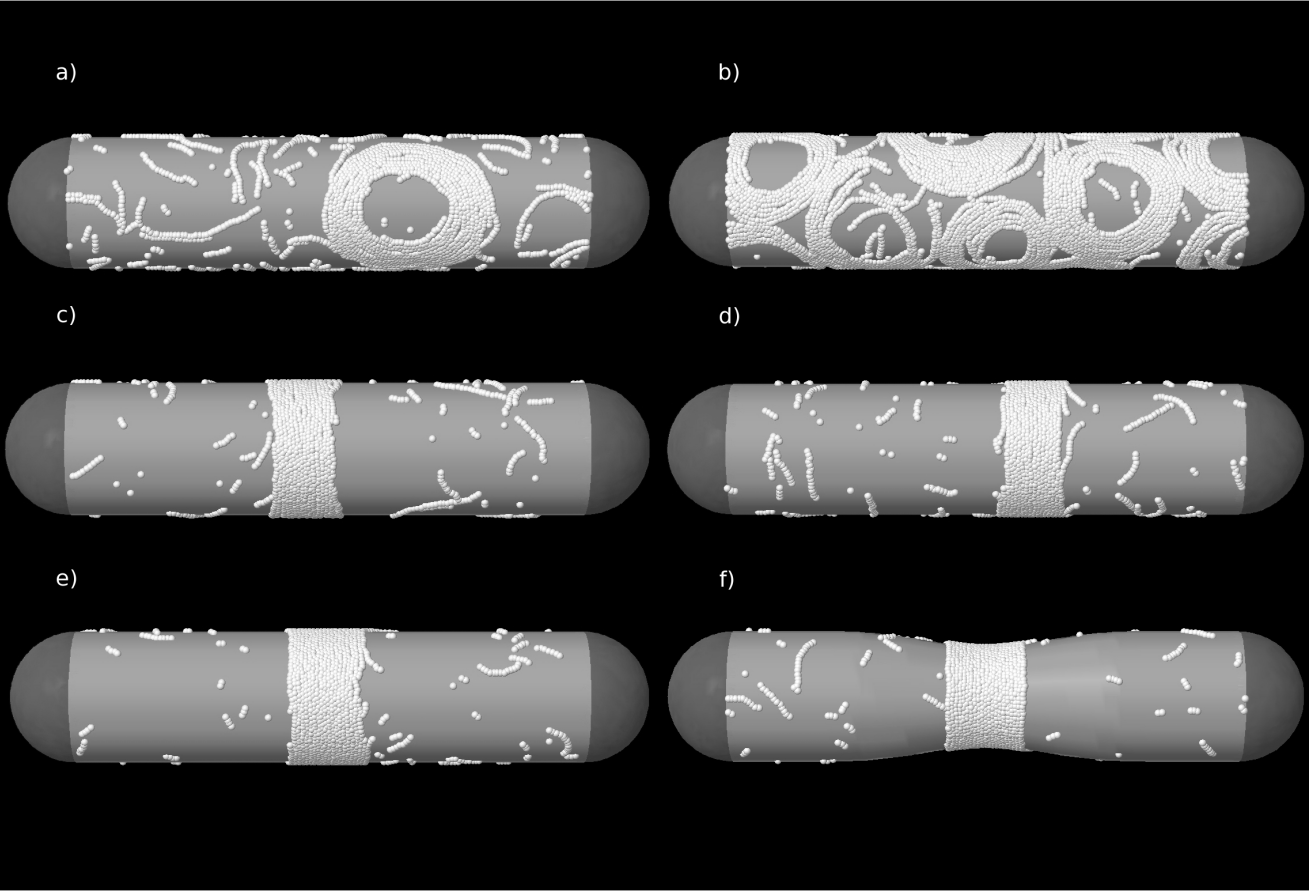
**Snapshots of equilibrated MC simulations on a cylinder**. Snapshots of equilibrated MC simulations on a cylinder. We use 10000 monomers in panel (b) and 2400 in all the other panels, which represents a surface density for FtsZ comparable to the one estimated in vivo [3]. (a-b) The interaction parameters extracted from AFM images on planar surfaces are directly used on the cylindrical lattice. (c) Symmetric bending energies *βU*_± _= 1, and *U*_*a *_and *U*_*b *_as in the planar case. (d) *U*_*a*_, *U*_+ _and *U*_- _are kept as in the planar model values and a weak off-plane spontaneous curvature is added (see file 1). (e) Bonding energy *U*_*b *_decreased by 2.5% away from a central cylindrical segment(see Additional file 1). (f) Similar to (e) but with a central deformation of a cylinder (see text and Additional file 1). All the observed structures were spontaneously formed from an initial random distribution of the monomers. The hemispherical caps are plotted to represent the bacterial shape, but the actual MC simulations were done with toroidal boundary conditions.

The main biophysical interest in modeling filament interactions on a surface is to extrapolate this understanding to explore their behaviour on a cylindrical surface comparable to bacterial geometry. This extrapolation requires extra assumptions for which we have no experimental information. We have explored different possibilities to see how generic or specific is the formation of cylindrical rings. The direct transfer of the planar lattice model parameters to a cylindrical geometry, without any off-plane spontaneous curvature, does not produce the formation of ring structures. In this case we assume that the polymer does not feel the curvature of the cylinder and that the energy of the bond would not change if a piece of the cylindrical surface containing that bond were cut along a line to flatten on a plane. As shown in Fig. 5(a-b) the filaments form rolls or open interconnecting structures similar to those observed on a planar substrate. The formation of ring structures requires, in addition to the condensation effects of lateral attractions [9,10], either the presence of a spontaneous off-plane curvature [15,16] or minimization of the on-plane curvature. The incomplete representations of the filaments in previous models prevented estimating the balance between both contributions but our fine-grained description now shows that tuning both, curvature and lateral interactions, can easily give rise to the formation of stable rings.

We first deal with modulations affecting filament curvature while the longitudinal and lateral interactions are directly extrapolated from the planar to the cylindrical geometry. Again, in this case we assume that the polymer does not feel the curvature of the cylinder. Eliminating the on-plane preferential curvature of the filaments by setting equal values for both bent bond penalizations allows the spontaneous formation of rings, as shown in Fig. 5(c). The results are quite robust with respect to the particular value of *U*_±_, which may be tuned to produce very flexible filaments (like in this figure with *U*_ ± _= *kT*) or much rigid ones (setting larger bending penalizations).

Alternatively, in Fig. 5(d) ring formation is also induced when adding an off-plane preferential curvature while keeping the on-plane curvature. Although we have no experimental data to quantify this off-plane energy, we can give an orientational dependence to the bond energy, and explore its effect using our lattice model. The result shown in the figure corresponds to a small bond modulation of Δ*U*_*b *_= 0.2 *kT*, over the planar bond estimate *U*_*b *_= 12 *kT*. This weak energetic bias in the alignment of the bonds is enough to overcome the tendency of the filaments to form rolls, and drives them to form ring condensates (see [11] and Additional file 1).

Affecting longitudinal bond energies can also condense the filaments into rings. Lowering filament longitudinal interactions outside a narrow cylindrical segment was used recently to simulate localized ring formation [9]. The effect was associated to the role played in vivo by the presence of MinC, known to induce filament shortening [27], in the cell regions where the ring is not formed. Longer FtsZ filaments would migrate to the MinC-free segment, where the FtsZ coverage would become very large. In ref. [9] the formation of the ring demands a drastic reduction of the energetic value of the longitudinal interaction from 17 *kT *= 10.2 *kcal/mol *to 3.6 *kcal/mol *under the assumed influence of the MinC system. The same concept applied to our fine-grained lattice model is much more efficient inducing the formation of rings and a small reduction of the bonding energy produces an equivalent effect. The ring in Fig. 5(e) is formed with the energy parameters obtained for FtsZ on mica, without any added off-plane spontaneous curvature and only reducing by 2.5% the bond energy outside the central part of the cylinder (Fig.S3 in Additional file 1 refers to the movies presented as additional files 2, 3, 4, 5, 6 that illustrate the formation of rings on a cylindrical surface for each of the conditions described in Fig. 5(a-e)).

The condensed aggregates formed under the effect of lateral attractions are driven towards a ring shape by any of the weak effects presented above. The rings obtained through the three alternative minor modifications (Fig. 5(c-e)) are formed as collections of parallel short filaments that actively exchange monomers and span only a fraction of the circumference. The parameters governing the FtsZ polymers on mica directly transferred to a cylindrical surface show that fine tuning the energetic balance confers the filament aggregates the high plasticity needed to form dynamic rings.

#### Z-ring contraction

Extrapolating the model to explain the formation of filament aggregates on a cylindrical surface also allows exploring possible force generating mechanism and quantitatively estimating the generated forces. The two interactions identified above as important in determining the ring forming capacity, lateral attraction between filaments and filament flexibility supporting a spontaneous curvature, have also been proposed as alternative force generating mechanism [9,16]. Our model indicates that in fact both could be relevant and there is no need to consider them as being mutually exclusive (see Additional file 1).

The radial force produced by single FtsZ filament ring kept around a cylinder by lateral attractions was previously evaluated within Langevin dynamics simulations with an off-lattice model [10]. We are now interested in exploring differences between the force generated by a long stabilized filament, and that of the Z-rings described here formed under GTP hydrolyzing conditions. These living polymers now retain their monomer exchanging capacity and form rings constituted by a collections of condensed disordered short filaments. We have used a mixed-approach in which the typical filament length distribution obtained with the triangular lattice model is transferred to the off-lattice model in ref. [10], for Langevin simulations of the equilibrated rings (see Additional file 1). The global structure of the ring is maintained by the attractive lateral interactions, and the distribution of the instantaneous radial force gives *F*_*R *_= 90(± 45)*pN*, i.e. slightly larger and more spread than in the single filament case (see Figure S4 and text in Additional file 1)). The force mechanism based on the lateral attractions, proposed and analyzed in ref. [10] for a single filament, is therefore not essentially modified by the multi-filamentary composition of the Z-ring. Within the calibration of that lateral interaction obtained from the experimental AFM data, the estimated radial forces fall within the correct order of magnitude to explain the observed deformation of the cylindrical liposomes under the action of FtsZ. Fig. 5(f) shows a central deformation of a cylinder similar to the one described in [7]. The figure was obtained from estimating the deformation of a multilamellar tubular vesicle subjected to the radial force of a ring formed when the bonding energy *U*_*b *_decreases by 2.5% away from the central cylindrical segment (see Additional file 1 for details).

## 3 Discussion

The lattice model presented here identifies the main interactions governing filament aggregate formation: a longitudinal interaction between monomers with preferential curvature and high flexibility and a lateral attraction between adjacent filaments. An interesting picture emerges from the quantitative estimate of their relative energetic values: the rich dynamic polymorphism including straight and curved condensates of various radius and densities observed experimentally can all be simulated from monomers diffusing on a two dimensional surface with a reduced set of parameters describing the basic interactions between FtsZ monomers. The comparison with the high resolution experimental results provided by the AFM images gives strong support to this minimal model that includes all the relevant interactions governing filament formation and condensation over a wide range of surface protein density.

A major conclusion of our analysis is that the interactions between the monomers are tuned to produce complex interconnecting structures formed by curved and straight filament bundles. Monte Carlo simulations of the lattice model with arbitrary values for the interaction parameters *U*_*a*_, *U*_*b *_and *U*_±_, show that in most cases the system would condense from a low density phase made of short polymers into a compact 2D nematic phase with parallel filaments, or into a compact meta-crystal made of very rigid spiral rolls(see Additional file 1). The polymorphic structures shown in Fig. 2 are obtained only under conditions where monomers exchange and a close balance between the spontaneous curvature and the lateral attraction of the filaments exists [25]. Coarser lattice model description of FtsZ [8,9] miss that balance and describe the FtsZ bundles as very rigid objects.

The fine balance between the energies governing monomer-monomer interactions produces very malleable polymers. An important consequence of this ductility is that, when formed on a cylindrical surface, as compared to a flat mica surface, small modulations can induce the spontaneous formation of rings. We have shown that at least three different conditions produce the same outcome: the reduction of the on-plane curvature, the existence of an off-plane spontaneous curvature, or a spatially localized weakening of the longitudinal bond are all enough to induce the formation of rings. Previous models predicted more stringent conditions for ring formation [9] and required the localized presence of MinC to originate rings. Our comprehensive model indicates, in agreement with available experimental evidence that indicates that MinC is not required to form rings ([28]), that alternative small perturbations in the interactions governing filament polymerization can also induce the formation of typical structures frequently observed in vivo: rings, under the conditions explained above, but also open helices when the lateral attractions are weakened, as is further described in [11]. Within the interaction parameters explored here, long filament bundles with helical orientation on the cylindrical membrane are observed as metastable structures. The movie S3-5c Fig.S3 presented in Additional file 4 shows their formation and their final adsorption by the stable ring structures.

The model perceives that reducing the spontaneous curvature observed on the mica surface or facilitating a small off-plane curvature would shift filament aggregates toward the formation of rings on a cylindrical surface. The on-plane spontaneous curvature observed can be easily canceled through different mechanisms: the formation of bundles of filaments with mixed plus-minus orientations ([25]and Additional file 1)or under any bundling promoting conditions [29-32]. An off-plane curvature could be associated to the presence of a long disordered segment in the membrane protein ZipA considered to be relevant in anchoring and bundling FtsZ filaments: the combination of reducing the on-plane curvature by bundling and providing a looser anchoring of the FtsZ protein to the surface facilitates the formation of rings on a cylindrical surface.

The rings observed in the MC simulations of our model are formed by short dynamic filaments and are essentially independent of the mechanism chosen to stabilize them Fig. 5(c-e). These predicted structures are similar to the incomplete filaments constituting the ring observed recently in *Caulobacter crescentus *[33] and their formation under monomer-monomer exchange conditions also agree with the observation that the rings formed in bacterial cells are capable of sustaining a dynamic monomer exchange [34,35].

It is remarkable that the simple model presented can account qualitatively for the most extensively described signature traits of FtsZ polymers: large polymorphism [29,36-42] formation of dynamic rings and helices on the bacterial cylindrical surface [33-35,43,44] and generation of a constriction force [7]. Furthermore, the plasticity described provides a rational explanation for the observed sensitivity of the polymer structure to precise experimental biochemical conditions: small modulations of monomer interactions can have a strong effect on the shape of the aggregates formed. The model also indicates that dynamic monomer exchange is required for the formation of condensed aggregates. This suggests that the essential role played by the GTPase activity in vivo could be related to maintaining the monomer dynamic exchange required to adopt the functional polymer aggregate conformation.

Recent experiments [7] showing the deformation of a tubular multilamelar liposome under the action of a modified form of the FtsZ protein provide the possibility of estimating from the observed deformation the minimal force required to produce such effect. Our estimate using the Helfrich hamiltonian model indicates the deformation requires a radial force of approximately 50*pN *(see Additional file 1). The *F*_*R *_= 90(± 45)*pN *generated by a dynamic condensed rings indicate that a contractile mechanism based on lateral attractions is robust and powerful enough to support the observed membrane contraction of a multilamelar liposome.

The debate between different alternatives for the formation of the Z-rings and the mechanism for the production of the constraining force has not been solved using coarse-grained models, which only incorporate a partial description of the FtsZ filaments. The model presented here is a minimal, but qualitatively complete, representation of this complex system and shows that the existing close balance between the curvature, flexibility and lateral attractions determine the plasticity of the FtsZ filament aggregates that can reconcile apparent contradictory models. We may speculate that such tuning of the *E. coli *FtsZ interactions, under active monomer exchange conditions for GTP-controlled polymerization, is related to the biological role of the protein: to form the septal ring at the correct time of the bacterial cycle and at the correct position on the bacterial membrane, driven by subtle bio-chemical signals.

## 4 Conclusion

In summary, this manuscript presents a model for FtsZ filaments formation extracted from the observation of the structure and the dynamic behaviour of individual FtsZ filaments imaged with atomic force microscopy. We observe that lateral interactions and curvature effects, both considered alternatively in previous models, are important to explain filament behaviour and, additionally, that their energetic balance is relevant to explain the rich polymorphism observed experimentally. The picture that emerges is that the FtsZ living polymers are malleable and very easily modulated to condense into contractile rings on a cylindrical surface.

The identification of the relevant monomer monomer interactions within the FtsZ filaments, their relative energetic contributions and the rich polymorphic behaviour supported by their finely tuned interactions constitutes a realistic and experimentally grounded account of the basic principles governing Z-ring contractile ring formation and represents a good starting point for future model refinements incorporating the effects of modulating agents that determine the positions, formation and contraction of the FtsZ ring in vivo.

## Supplementary Material

Additional file 1 **Supplementary material**. The text, including 3 figures, corresponds to the supplementary material to the main text of the article. Click here for file

Additional file 2 **First movie out of 5 that correspond to figure S3 in supplementary material**. MC simulation on a cylinder using the conditions described in Fig. 5a of the main text. Click here for file

Additional file 3 **Second movie out of 5 that correspond to figure S3 in supplementary material**. MC simulation on a cylinder using the conditions described in Fig. 5b of the main text. Click here for file

Additional file 4 **Third movie out of 5 that correspond to figure S3 in supplementary material**. MC simulation on a cylinder using the conditions described in Fig. 5c of the main text. Click here for file

Additional file 5 **Fourth movie out of 5 that correspond to figure S3 in supplementary material**. MC simulation on a cylinder using the conditions described in Fig. 5d of the main text. Click here for file

Additional file 6 **Fifth movie out of 5 that correspond to figure S3 in supplementary material**. MC simulation on a cylinder using the conditions described in Fig. 5e of the main text. Click here for file
