# Supplementary material for: Simple modeling of FtsZ polymers on flat and curved surfaces: correlation with experimental in vitro observations
Source: PMC Biophys. 2009 Oct 22;2:8. doi: 10.1186/1757-5036-2-8 (PMC2776577; doi:10.1186/1757-5036-2-8)
Supplement: Additional file 1 — Supplementary material. The text, including 3 figures, corresponds to the supplementary material to the main text of the article. [file 1757-5036-2-8-S1.PDF]

Simple modeling of FtsZ polymers on flat and curved surfaces: correlation with experimental in vitro observations.

Supplementary Material

Email:

*Corresponding author

Planar lattice model

We first establish, as in any Monte Carlo (MC) simulation in statistical physics, a representation of all the possible states of system, the energy associated to any of these states, and the set of rules used to select trial moves between different states [1]. Previous lattice models for FtsZ [2, 3] have used a coarse description for the position of the monomers on the bacterial membrane, with square-lattice representations in which each cell has the size of a protein monomer, $d \approx 4.5nm$. To incorporate the structural features observed in the AFM images, we use finer-grained description with a two dimensional triangular lattice of sites separated by $\delta r = 1.5nm$. Each monomer excludes to the other monomers its own lattice position and another $30 \approx \pi(d/\delta r)^2$ neighbor sites, as shown in Fig.S1. We assume that the direction from the *minus end* (T7 loop-region) to the *plus end* (GTP binding site) is parallel to the mica surface, and we represent the monomer orientation as a discrete set of 24 directions.

A crown of 42 lattice sites, around the excluded core (see Fig. S1) gives the relative lattice positions where two monomers, independently of their orientations, get an attractive energy $-U_a$, which provides the *lateral attraction* between the filaments. The stronger longitudinal *bonding interaction* which produces the filaments applies only when the polarization vectors of two polymerized monomers are equal or differ in one step (15 degrees). The energies $-U_b$, $-U_b + U_+$ and $-U_b + U_-$ correspond to the only three allowed cases: straight, one clockwise step difference and one anti-clockwise step difference.

Besides this restriction in their orientations, bond formation requires that two interacting

monomers are located within a reduced set of lattice positions, as shown in Fig. S1. When the two monomers have the same orientation there are 4 or 5 lattice positions at which they will have the full bonding energy $-U_b$. When their orientations differ in one step, clockwise or anti-clockwise, the restriction is stronger, because the relative position vector has to be within 30 degrees of both polarizations, and only 3 or 4 lattice positions are available to form a bond. In this case the energy is reduced to $-U_b + U_+$ or $-U_b + U_-$, depending on the whether the relative orientation of the two monomers is right or left.

A complete description of the lattice model is made in ref. [4]. A technical but important property of our model is that it uses only pairwise interactions, i.e. the interaction energy between two monomers depends only on their orientations and relative position, and it is independent of whether or not each monomers forms part of a filament. Pairwise models are much easier to simulate, but in flexible *living polymer* models they may produce the spurious branching of the filaments. The large excluded core region, compared with the narrow bonding regions, avoids this problem while retaining enough bonding sites to allow the changes in the filament shape through individual moves of the monomers.

The typical length of the filaments is determined by the value of U_b, while the parameters U_+ and U_- allow to tune their rigidity and spontaneous curvature, and U_a their tendency to aggregate in condensed structures. However, all these effects are entangled, and the same set of parameters produces different structures depending on the protein coverage of the surface.

Monte Carlo simulations

The definition of our lattice model, with a few relative positions compatible with the formations of bonds, allows an effective sampling of the configurations on the basis of elementary moves of a monomer to any of the six neighbor lattice positions, and rotations to one of the two adjacent orientations. These *local* motions and rotations give a qualitative representation of the brownian motion of the FtsZ monomers on the surface. To accelerate the convergence to thermal equilibrium we have also considered *global* moves in which a monomer is moved to a randomly chosen site all over the lattice, that may represent the desorption to the bulk solution, followed by the re-adsorption at a different place. In any case we use the standard Metropolis algorithm to accept of reject the trial MC moves [1]. Typical MC simulations were run on 607×607 lattice sites, with periodic boundary conditions and with a fixed number of monomers between 400 (very low coverage)to 8000 (very high coverage). Starting from randomly chosen positions and orientations of the monomers the system was equilibrated over 10^7 MC attempts to move or rotate each monomer, and then sampled over $\sim 3 \times 10^7$ MC attempts per monomer to get the statistical equilibrium properties and the

representative snapshots of the equilibrated system.

Estimation of model parameters for FtsZ-on-mica

MC simulations of the lattice model with different values for the interaction parameters, relative to the thermal energy $kT \equiv 1/\beta$, produce strongly polymorphic aggregates and textures (see Fig. S2). Long filaments are formed when $\beta U_b \geq 10$, while lower lateral attractions, $\beta U_a \leq 1$, keep a wide gap between the two hierarchical levels of organization: monomers form filaments and filament form aggregates. A symmetric choice $U_+ = U_- \ll U_b$ produces very flexible filaments with no preferential curvature, whereas large symmetric values $U_+ = U_- \approx U_b$ would produce very stiff and straight filaments. The spontaneous curvature observed in AFM images for FtsZ on mica requires asymmetric values, which we (arbitrarily) take as $U_+ \gg U_-$, since the plus-minus direction of the filaments cannot be distinguished in the experimental results.

A bond energy of $U_b = 12(\pm 2)kT \approx 7.2 kcal/mol$, a small left-bent penalization $U_- = 0.5(\pm 0.5)kT$, and $U_+ = U_b$ to cancel the right-bent bond energy can describe the observed shape and length of the filaments at low protein coverage. A lateral attraction of $U_a = 0.5(\pm 0.1)kT \approx 0.3 kcal/mol$ determined the aggregation of isolated clusters. The resolution of the experimental technique and the simplicity of the model do not allow a more precise tuning of the interaction energies. However, the model represents a qualitative improvement over any previous lattice model description of FtsZ, since the four interaction parameters are selected from the direct experimental evidence of the filament structures observed with AFM. The good match between the characteristics observed in the equilibrated snapshots of the lattice model and the AFM images covers the full range of protein coverage with the same set of interactions parameters.

On the other hand, it is important to realize that the above given values for the model interaction parameters, chosen to give the best representation of *E. coli* FtsZ on mica, appear to be tuned to give very malleable structures. Most of the possible choices for $U_\pm$ would produce either tightly rolled structures, as those in Fig. S2(a), or straight filament bundles as those in Fig. S2(c). The coexistence of curved and straight bundles as those in Fig. S2(b) requires a fine tuning of the bent bond energies and the lateral interaction, and it produces polymorphic structures which may be driven to very different forms under the effect of very small bias.

Lattice model on a cylindrical surface

The formation of rings on a cylindrical surface using the coarser square-lattice models for FtsZ [2, 3] was accomplished by including either off-plane spontaneous curvature or the bond weakening effect of the MinC system to confine the condensation of the filaments into a ring. The triangular-lattice model presented in this work can also be rolled into a cylindrical shape (along any of the symmetry directions of the lattice). The structures of the FtsZ filaments formed on such curved surfaces present a qualitatively new feature not visible in the planar geometry: filaments, or bundles of filaments, go around the cylinder and form Z-ring structures [5].

Alternative assumptions for the curvature energy

The optimal bond energy U_b, the lateral attraction energy U_a, and the excluded area effects can be directly transferred from the planar to the cylindrical lattice. However, the curvature effects are more complex since filaments on a cylindrical support have three different bending modes: on-plane bending, twist and off-plane bending, each with its rigidity and preferential angle [6]. The on-plane bending mode is a "biased flexibility" with three types of bonds (left-turn, straight and right-bent) with different (and asymmetric) bonding energies. These same bonds are included in our two-dimensional model describing the bending of filaments on a plane, so the information gained from the AFM images of FtsZ on mica can be directly used to calibrate the bending rigidity ($\kappa^{\text{on-p}}$) and spontaneous curvature ($H_o^{\text{on-p}}$) of this mode.

To that effect we have run short MC simulations of single filaments with permanent bonds (i.e. limit $U_b \gg kT$) and in absence of lateral attraction ($U_a = 0$), but with the same U_+ and U_- values as obtained from the global comparison of the AFM images and the MC simulations in [4]. The shape of these filaments was then fitted to polynomial forms in polar coordinates, as explained in [5], and the curvature histograms were then used to obtain the filament rigidity and the mean curvature.

The second (twist) mode on a cylinder would favor helical structures with a preferential pitch, and is not contemplated here. The third (off-plane bending) mode, whose effect we do want to consider, would imply a preferential radius for the cylindrical support. Since the two-dimensional treatment cannot deal with the off-plane bending, a different treatment is applied. We consider that the off-plane curvature is imposed by the geometry of the underlying lattice, not associated to any off-plane flexibility, so that we only have to look for the relative direction of the two monomers, with respect to the direction of the cylindrical axis, to give the penalization/enhancement of their bond. A free energy contribution per monomer

(of diameter d)

$$F_{\text{off-p}} = \frac{d\ \kappa^{\text{off-p}}}{2} \left(H - H_o^{\text{off-p}}\right)^2, \tag{1}$$

where H is the inverse of the cylindrical radius when the filaments forms a ring, and $H = 0$ when oriented parallel to the cylindrical axis. The minimization of (1) for a fixed cylindrical radius $R > 1/H_o^{\text{off-p}}$ would lead to the orientation of the filament in the plane normal to the cylinder axis, favoring the formation of rings. Moreover, if (1) is also minimized with respect to the radius of the cylinder, it would push R down towards the optimal filament radius $1/H_o^{\text{off-p}}$.

Unfortunately, we have no direct experimental data for the twist and off-plane bending modes of FtsZ. In the analysis for single long filaments with no lateral attraction [6], the formation of rings was associated to a very specific condition of null spontaneous pitch. However, it was later shown [7] that the effects of the lateral attractions between the filaments would easily produce tight helical rings independently of the preferential pitch of the bonds. Therefore, any effect of the second and third bending modes on a cylinder would get mixed into the effective off-plane $\kappa^{\text{off-p}}$ and $H_o^{\text{off-p}}$ to be used in (1). This curvature effect has been proposed by several authors as the driving force for the formation of Z-rings and the production of the constraining force on the cylindrical membrane [8–12]. Within our cylindrical lattice model for FtsZ, we may include the off-plane bending mode as a modulation of the bonding energy U_b, which becomes a function of the angle φ between the direction of the bond and the cylindrical axis. At the leading order for $\Delta U_b \ll U_b^{\text{planar}}$ we may write

$$U_b(\varphi) = U_b^{\text{planar}} - \Delta U_b\ cos(2\varphi), \tag{2}$$

where the comparison with (1) gives $\Delta U_b = \kappa^{\text{off-p}} d(2RH_o^{\text{off-p}} - 1)/(4R^2)$.

As said above, the planar AFM images of FtsZ on mica provide only information on the on-plane mode, tuned by the model parameters $U_\pm$ and producing the typical planar spirals. There is no direct experimental data for the off-plane rigidity $\kappa^{\text{off-p}}$ and spontaneous curvature $H_o^{\text{off-p}}$, and the most educated guesses come from the direct extrapolation from the on-plane curvature of filaments on planar substrates, observed with AFM [5] or electron microscopy [13]. The observed long FtsZ filaments on mica, linked with modified GDP-Al_3 nucleotide (to reduce the hydrolysis at the bonds) [5] gives $\kappa^{\text{on-p}} \approx 1.6 \times 10^4 pN(nm)^2)$ and $1/H_o^{\text{on-p}} = R_o \approx 65nm$. However, it is known that the flexibility of protein filaments may be strongly affected by the biochemical environment, and there are some evidences much lower rigidity modulus for FtsZ filaments under different conditions [13]. Moreover, there could be important differences between the bending modes of FtsZ filaments tightly anchored to a solid substrate and those with a loosely linked (through peptide chain) to a bilayer membrane. The flexibility of the link could favor the bundling of filaments, leading to larger effective rigidity but null spontaneous curvature [14].

Altogether, in our MC simulations for the lattice model with cylindrical geometry we have explored three different possibilities regarding the curvature effects on a cylindrical substrate:

i) The direct transfer of the on-plane spontaneous curvature from the planar AFM experiments of FtsZ on mica, included through the values of the $U_{\pm}$ bent bond parameters, and neglecting any spontaneous pitch and off-plane curvature.

ii) The assumption (see ref. [7]) that under a looser anchoring of the FtsZ to the bacterial membrane, the formations of filament bundles leads to the cancellation of all the spontaneous curvatures, through a choice of $U_+ = U_-$ in our lattice model and neglecting any off-plane curvature effect.

iii) The assumption that on top of the on-plane spontaneous curvature extracted from the experimental planar system, we add the effect of an off-plane curvature as a weak bias (2) on the orientation of the filaments. In this case we have used the estimates given above for the direct transfer of the on-plane bending constants $\kappa^{\text{off-p}} \approx \kappa^{\text{on-p}}$ and $H_o^{\text{off-p}} \approx H_o^{\text{on-p}}$, and taking the E. coli radius $R \approx 500nm$, get from (2) $\Delta U_b \approx 0.25kT$ at room temperature. That is probably an overestimation for the possible off-plane curvature effects, and still it is a small ($\pm 2\%$) bias with respect to the bonding energy in the planar system $U_b = 12kT$.

As described in the main article of this supplementary material, the spontaneous formation of Z-rings, under the driving force of the lateral attraction between FtsZ filaments, is observed under assumptions *ii*) and *iii*), but not under *i*).

Spatial modulation of the bonding energy by the MinC system

The recent proposal for the formation of FtsZ rings in a coarse cylindrical lattice model [3] without any curvature effect, invokes the role of the MinC system to provide a spatial modulation of the interactions between FtsZ monomers. The *raw* interactions ($\epsilon_1 = 17kT$, playing the role of our U_b, and $\epsilon_2 = 0.2kT$ playing for our U_a parameter) act only on a small cylindrical segment. Away from that segment the interactions are strongly reduced to $\epsilon_1 = 6kT$, and $\epsilon_2 = 0$, through the assumed effect of the MinC system.

Since the model in ref. [3] includes no curvature effect, we may compare it with our finer-grained model under the assumption *ii*) in the previous subsection. Therefore, our model demonstrates that, for flexible filaments with no spontaneous curvatures (or with a moderate off-plane spontaneous curvature), the lateral attraction is enough to explain the self-assembly of FtsZ rings, without any need of a MinC-system. Under the assumptions *ii*) and *iii*) of the previous section, the only qualitative effect of assuming a spatial modulation of U_b would be to

locate the Z-ring at the position were FtsZ forms the stronger bonds, instead of getting the rings formed at random positions along the MC simulations. The coarse lattice model presented in ref. [3] however requires the strong assumed effect of the MinC system to get the formation of the Z-ring.

We may explore in our case the efficiency of the MinC-effect when the Z-ring is not spontaneously formed, like in the above assumption i). To that effect we have run MC simulations, keeping the on-plane tendency to form spiral cluster and without any off-plane curvature, when we consider that the bonding energy U_b is (slightly) reduced for bonds away from a Min-C free cylindrical segment.

The high malleability of the structures formed in our fine-grained lattice model, under the direct transfer of the monomer interaction obtained from FtsZ on mica, shows up in the formation of Z-rings under a very weak bias. In particular, a mere 2.5% depletion of U_b (from $7.20 kcal/mol$ to $7.02 kcal/mol$) was shown to be enough to form the rings under the unfavorable assumption i) of the previous section. In comparison, the strong change (from more that $10 kcal/mol$ to only $3.6 kcal/mol$) required in ref. [3] should be regarded as an artifact of the coarse-grained description in that model, which fails to capture the structural softness of the FtsZ aggregates.

In fact, the strong MinC-effect assumed in ref. [3] would require an unphysically large rigidity for MinC-FtsZ complex. On biochemical grounds, the depletion of the FtsZ bonding energy U_b in more than $6 kcal/mol$ would imply a similar affinity between FtsZ and some of the MinC system proteins. This would so block the formation of FtsZ filaments. Therefore, to keep that MinC element away from the Z-ring region the MinC system should be able to stand a free energy drop of $\sim 6.4 kcal/mol$ over a distance of one monomer size, i.e. each MinC monomer should stand against a force of $\sim 2.3 kcal/nm \approx 10 pN$. In mechanical terms, the formation of the Z-ring in that coarse lattice model needs the assumed MinC effect to compress all the FtsZ into a narrow cylindrical segment, and such compression requires that the MinC system acts as a very rigid piston.

In contrast, with our finer-grained lattice model the Z-rings are spontaneously formed under favorable assumptions (ii) and iii)), and even under the unfavorable assumption i), if a mild modulation of the FtsZ bonding free energy is present. The 2.5% depletion of U_b could be achieved with less than $0.3 pN$ force on the proteins forming the MinC system at the edge of the Z-ring. This force could be further reduced if a smooth spatial modulation of U_b, instead of the sharp-step profile used in ref. [3], is considered.

Fig.S3 refers to the movies presented as Additional files 2-6 that illustrate the formation of rings under the different conditions described in Fig.5 in the main text.

Z-ring contraction and membrane deformation

Recent experiments [15] have given the first experimental in vitro evidence for the deformation of a tubular liposome under the action of a modified form of the FtsZ protein which anchors on the inner bilayer membrane of multilamellar cylindrical liposomes. The observed shape of the lamellar tubes may be used to estimate the force done by the ring, and to compare it with the estimation of the ring force produced by model descriptions of the FtsZ filaments.

Helfrich hamiltonian for the membrane shape

The deformation of a cylindrical membrane under the action of a force-ring may be analyzed within the Helfrich hamiltonian model [16], in terms of the surface tension σ, the spontaneous mean curvature H_o, and the bending rigidity κ. The membrane free energy is given by the area integral

$$F_{\text{mem}} = \int dA \left[\frac{\kappa}{2} (H - H_o)^2 + \sigma \right], \tag{3}$$

Where H is the local curvature of the surface, i.e. $H = 1/R_{\text{u}}$ for an undeformed cylinder and

$$H = \frac{1}{R_{\text{mem}}(z)(1 + R'_{\text{mem}}(z)^2)^{1/2}} - \frac{R''_{\text{mem}}(z)}{(1 + R'_{\text{mem}}(z)^2)^{3/2}} \tag{4}$$

for a deformed surface with axial symmetry described by the radius $R_{\text{mem}}(z)$ as a function of the z coordinate, along the axis.

The minimization of (3) under the assumptions $R_{\text{mem}}(z) \to R_{\text{u}}$ for $z \to \pm\infty$, and $R_{\text{mem}}(0) = R_{\text{ring}}$, gives the shape of a tubular membrane under the constraint of a force-ring of radius $R_{\text{ring}} < R$. That (conditional) minimal shape may then be used to evaluate the minimal free energy (3) as a function of R_{ring}, and the derivative $dF_{\text{min}}(R_{\text{ring}})/dR_{\text{ring}}$ gives the radial force done by the ring.

The analysis within the linearized Helfrich hamiltonian of the large multilamellar tubular forms in ref. [15] indicate that the observed deformation requires a radial force of approximately $50pN$ [17]. We may now ask under which assumptions that force may be produced.

Estimations of the Z-ring force

The analysis of the possible force-generating mechanisms is subject to strong uncertainties. The two proposed mechanisms are the same as those proposed for the formation of the Z-ring: The condensation induced by the lateral attraction between the filaments and the possible

spontaneous curvature of the phospholipid bilayer induced by the anchored FtsZ filaments. In fact none of these mechanisms excludes the other. We have seen that a spontaneous off-plane curvature is one of the possible modifications of the planar lattice model parameter for FtsZ on mica that may produce the formation of rings in our model. On the other hand, the lateral attraction between filaments expand the parametric space for with spontaneous curvatures on a cylindrical support lead to the formation of tight spiral rings in long filaments [7].

Let us consider first the off-plane spontaneous curvature as the possible origin of the contractile force. With the estimates used above, i.e. taking the measured on-plane rigidity and spontaneous curvature [5] as a guess for the parameters of the off-plane bending mode, $\kappa^{\text{off-p}} \sim 1.6 \times 10^4 pN(nm)^2$ and $1/H_o^{\text{off-p}} \sim 65nm$, we may estimate the constraining force on the large multi-lamellar cylindrical liposomes in ref. [15] to be about $5 \times 10^{-3} pN$ per FtsZ monomer. Therefore, the required $50pN$ needed to produce the deformation of the tubular liposome could be achieved with 10^4 monomers, i.e. with a relatively narrow ring of $4-$filament width. However, there are much lower estimates for the rigidity of the single FtsZ filaments, which would push that force estimate to less than $3 \times 10^{-4} pN$ per FtsZ monomer. The formation of filament bundles, under a looser anchoring of the FtsZ monomers to the surface, could enhance the rigidity, but at the same time it would most probably cancel the spontaneous curvature [14], so that the curvature-induced force would disappear.

Now we turn to the possible role of the lateral attraction between the filaments, as the driving force to create the constriction. The evaluation of the radial force done by single FtsZ filament ring has been evaluated within Langevin dynamics simulations with an off-lattice model [5]. The use of such continuous model is both easier and more accurate than lattice models for the evaluation of force, while the breaking and remaking of the strong bonds along the filaments is computationally difficult in continuous models. Therefore, we have used a mixed-approach in which the typical filament length distribution obtained with the triangular lattice model is transferred to the off-lattice model in ref. [5], for Langevin simulations of the equilibrated rings. In that work the force was obtained for long single filaments kept by the lateral attractions in tight helical structures around a cylinder. Here we want to consider the possible differences between the force of single filaments, and that of Z-rings formed by disordered collections of condensed shorter filaments.

In a continuous model the radial force may be obtained by the usual virial method [1], i.e. the average over thermal equilibrium configurations of the derivative of the Z-ring energy with respect to a global expansion of the cylindrical support,

$$F_R = - \left\langle \frac{\partial U(\mathbf{r}_1, \mathbf{r}_2, ..., \mathbf{r}_N)}{\partial R} \right\rangle, \tag{5}$$

when the positions of the N monomers are written in cylindrical coordinates as

$$\mathbf{r}_i = (Rcos(\varphi_i), Rsin(\varphi_i), z_i), \tag{6}$$

and the derivative with respect to R is done keeping constant all φ_i and z_i. This method gives the the full radial force, including the entropic terms which were neglected for the structures of a single filament in ref. [5].

The results in Fig. S4 show the instantaneous value of the radial force and its probability distribution, taken over 1000 time units of the Langevin simulation, which was estimated to be in the range of $t_o = 0.1$ seconds. The top curve corresponds to a single filament with 250 monomers, in an helical structure with 5 full rounds around a cylindrical support of $40nm$ of radius showing instantaneous radial force values $F_R = 80(\pm 30)pN$, following a gaussian distribution. Notice that the fluctuations around the mean force are rather large, and correlated over times of the order of $10^2 t_o$.

The lower panel n Fig. S4 shows the results for the radial force when the same number of monomomers (250) area split in 15 filaments, within the typical size distribution observed for Z-rings in the lattice model. The global structure of the ring is maintained by the attractive lateral interactions, and distribution for the instantaneous radial force gives $F_R = 90(\pm 45)pN$, i.e. slightly larger and more spread than in the single filament case. The most important difference is the absence of the long time correlations observed in the single filament case.

Altogether, it is clear that the force mechanism based on the lateral attractions, proposed and analyzed in ref. [5] for a single filament, is not essentially modified by the multi-filamentary composition of the Z-ring. Within the calibration of that lateral interaction obtained from the experimental AFM data, we predict radial forces within the correct order of magnitude to explain the observed deformation of the cylindrical liposomes under the action of FtsZ.

References

1. Dan Frenkel, Daan Frenkel, and Berend Smit, Understanding Molecular Simulation: From Algorithms to Applications, 2002, Academic Press

2. Lan,G., Dajkovic,A., Wirtz,D. and Sun,S.X., Polymerization and Bundling Kinetics of FtsZ Filaments,Biophys J.,2008.

3. Ganhui Lan, Brian R. Daniels , Terrence M. Dobrowsky , Denis Wirtz , and Sean X. Sun Condensation of FtsZ filaments can drive bacterial cell division, PNAS 2009, 106, 121-126

4. Paez,A, Mateos,P., Vélez,M. and Tarazona,P., Self-organization of living polymers: Adsorbed protein filaments, Soft-Matter 2009,5, 26252637

5. Hörger,I., Velasco,E., Mingorance, J., Rivas, G. Tarazona, P. and Vélez, M.Langevin computer simulations of bacterial protein filaments and the force-generating mechanism during cell division ,Physical Review E,2008,77,011902

6. Andrews, S. S., and A. P. Arkin. A mechanical explanation for cytoskeletal rings and helices in bacteria ,Biophys. J ,2007,93,1872-1884.

7. Hörger,I. , Velasco,E. ,Rivas, G., Vélez, M. and Tarazona, P. FtsZ Bacterial Cytoskeletal Polymers on Curved Surfaces: The Importance of Lateral Interactions, Biophys. J.,2008, 94,L81-L83

8. Huecas,S., Llorca,O., Boskovic,J., Martí n-Benito, J., Valpuesta, J..M., and Andreu,J.,Energetics and Geometry of FtsZ Polymers: Nucleated Self-Assembly of Single Protofilaments , Biophysical J., 2008, 94,1796-1806.

9. Miraldi,E., Thomas, P.J. and Romberg,L., Allosteric Models for Cooperative Polymerization of Linear Polymers, Biophys. J. ,2008, doi = 10.1529/biophysj.107.126219.

10. Surovtsev, I.V., Morgan,J. J. and Lindahl,P. A.,Kinetic Modeling of the Assembly, Dynamic Steady State, and Contraction of the FtsZ Ring in Prokaryotic Cytokinesis,PLoS Comput Biol,2008, 4,e1000102,doi = 10.1371/journal.pcbi.1000102

11. Ghosh,B. and Sain, A.,Origin of Contractile Force during Cell Division of Bacteria PRL 2008,101, 178101

12. Jun F. Allard and Eric N. Cytrynbaum, Force generation by a dynamic Z-ring in Escherichia coli cell division PNAS, 2009,106 , 145-150

13. Dajkovic,A., Lan, G., Sun,S.X., Wirtz,D. and Lutkenhaus,J., MinC Spatially Controls Bacterial Cytokinesis by Antagonizing the Scaffolding Function of FtsZ,Current Biology, 2008, 18,235-244

14. to be published.

15. Osawa, M., Anderson, D. and Erickson,H . , Reconstitution of Contractile FtsZ Rings in Liposomes ,Science,2008, 320, 792

16. Helfrich W., Z. Naturforsch, 1973, 28c, 693.

17. to be published

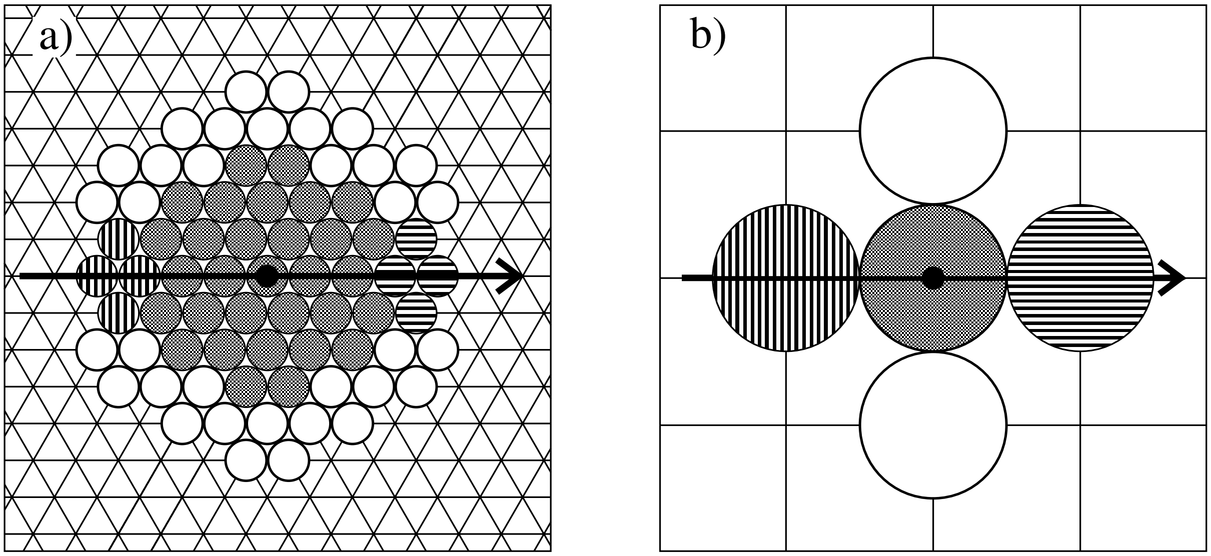

Figure 1: Panel (a) shows the triangular lattice model used here, in which a monomer at the central position (small black circle) excludes the occupation of any of the 31 (grey circle) positions around it, and gets attractive interactions with those at any of the 42 surrounding sites (white or barred circles). Within this crown of attractive positions, those marked by horizontal bars indicate the allowed position for the formation of a strong longitudinal bond between two monomers with the orientation indicated by the arrow. Monomers with the opposite orientation would form a strong bond when their relative position is within the set with vertical bars. Similar choices of 4 or 5 bonding sites are selected for any of the 24 allowed orientations (see text and ref. [4]). Panel (b) represents, at approximately the same real scale, the coarser square-lattice model used in ref. [3], in which each monomers excludes others only from its own lattice position, the attractive crown is restricted to the 4 nearest neighbor sites, and there is a single bonding site for each of the 4 allowed orientations.

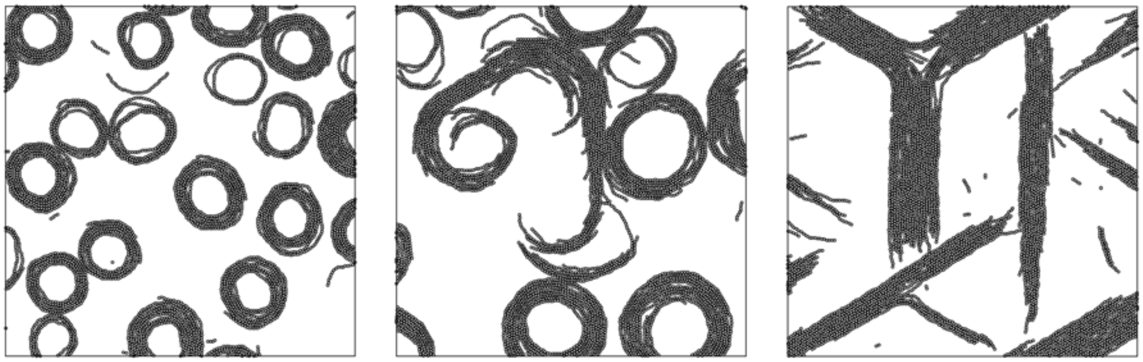

Figure 2: Snapshots of equilibrated configurations, taken along MC simulations of our model with different values of the bent-bond energies. The protein coverage ($2picoMol/cm^2$), the straight bond energy ($U_b = 12kT$), and the lateral attraction ($U_a = 0.5kT$) are the same in the three cases. Panel (a) corresponds to $U_+ = U_b$ (full cancellation of the bond energy for the + bent), and $U_- = 0$ (no penalization of the − bent). Panel (b) comes from the best fit to FtsZ on mica, $U_+ = U_b$ and $U_- = 0.5kT$. Panel (c) uses the symmetric choice $U_+ = U_- = 2kT$.

Figure 3: The five movies represent MC simulation on a cylinder using the same conditions described in Fig.5 of the main text. We use 10000 monomers in movie S3-5b and 2400 in all the others. In the movies S3-5a and S3-5b the interaction parameters extracted from AFM images on planar surfaces are directly used on the cylindrical lattice. The movie S3-5c is obtained using symmetric bending energies $\beta U_{\pm} = 1$, and U_a and U_b as in the planar case. In movie S3-5d U_a, $U_+$and U_- are kept as in the planar model values and a weak off-plane spontaneous curvature is added. In movie S3-5e the bonding energy U_b decreased by 5% away from a central cylindrical segment to simulate the effect of MinC

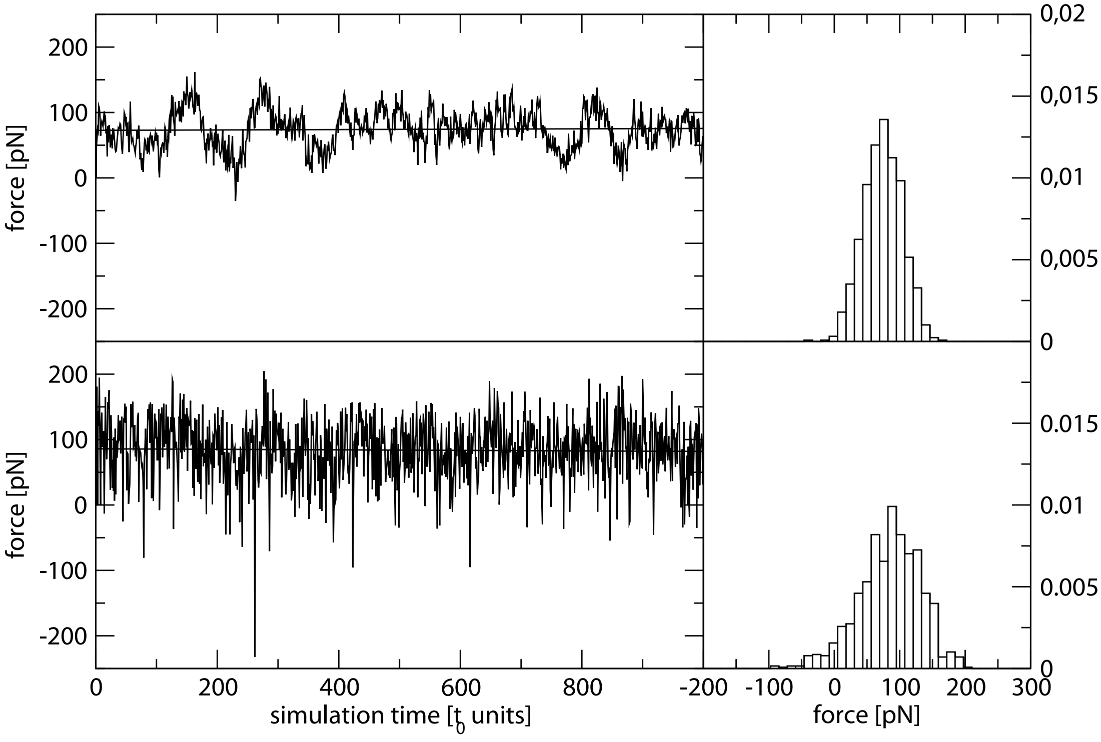

Figure 4: The instantaneous radial force produced by 250 monomers in a Z-ring formed by a single filament (top) or 15 filaments (bottom), on a cylinder of $R = 40nm$, along 1000 times steps of the Langevin simulation of the continuous model in ref. [5]. On the right had side the histograms for the distribution of force values.
